# Effects of walnut green hsuk on the quality, bacterial community diversity and *in vitro* rumen digestion characteristics of whole-plant corn silage

**DOI:** 10.3389/fvets.2025.1720310

**Published:** 2026-01-14

**Authors:** Naibi Abulaiti, Fangxia Wang, Aibibula Yimamu

**Affiliations:** Key Laboratory of Grassland Resources and Ecology of Xinjiang, College of Grassland Science, Xinjiang Agricultural University, Urumqi, China

**Keywords:** walnut green husk, whole-plant corn, silage quality, *in vitro* nutrient digestibility, digestive enzyme activity, bacterial community diversity

## Abstract

This experiment aimed to investigate the effects of adding walnut green husk (WGH) on the quality of whole-plant corn silage, bacterial community diversity, and *in vitro* rumen fermentation characteristics. The silage was prepared using whole-plant corn (Qidan 828) at the milk-ripening stage and walnut green husk (WGH, Xinwen 185) as raw materials. Four treatments were established: CK (100% whole-plant corn), A1, A2, and A3, with WGH added at rates of 15, 30, and 45 g per kg of fresh matter, respectively. Each treatment was ensiled in laboratory-scale silos for 60 days under dark and anaerobic conditions at 16 °C−22 °C, with three replicates per group. The results indicated that WGH supplementation significantly elevated the DM and CP content and concurrently lowered the levels of acetic acid, propionic acid, and NH_3_-N (*P* < 0.05). Consequently, it increased the lactic acid bacteria population and reduced the yeast count (*P* < 0.05). This microbial shift ultimately led to improved aerobic stability. Compared to the CK group, adding WGH reduced the abundance and diversity of the microbial community. At the phylum level, significant shifts in microbial composition were observed. The relative abundance of *Firmicutes_D* was higher in the A2 and A3 groups than in the control. In contrast, the abundances of *Bacteroidota, Proteobacteria*, and unclassified bacteria were significantly reduced in these treatment groups. At the genus level, we observed an increase in the abundance of beneficial genera, including *Lactiplantibacillus, Levilactobacillus*, and *Lacticaseibacillus*. Thisenrichment of lactic acid bacteria significantly improved the overall bacterial community structure of the whole-plant corn silage. Among these, the A2 treatment had the highest relative abundance of dominant bacteria *Firmicutes_D* (82.79%), *Lactiplantibacillus* (66.48%), *Levilactobacillus* (14.03%), and *Lacticaseibacillus* (4.22%). During *in vitro* rumen fermentation, increasing the WGH ratio elevated the activity of all measured digestive enzymes except for protease. This enhancement further led to significant increases in IVDMD, IVCPD, and IVNDFD (*P* < 0.05). However, a significant decrease was observed in IVADFD (*P* < 0.05). This study confirms the feasibility of the functional utilization of WGH as an unconventional feed resource for enhancing silage quality by driving beneficial microbial shifts. Therefore, an inclusion rate of 30 g·kg^−1^ is recommended as the most appropriate to achieve optimal fermentation and digestibility. Future efforts should be directed toward practical application at scale and elucidating the modulatory mechanisms.

## 1 Introduction

Whole-plant corn (*Zea mays L*.) is a high-yielding, nutritious, and palatable forage. It is widely used as a premium roughage in cattle feed and a key raw material for silage. This has established it as a crucial component supporting the rapid development of ruminant animal husbandry (1, 2). In recent years, with the comprehensive implementation of China's “fodder substitution for grain” project, the planting area of silage corn has gradually expanded and been widely used in ruminants, thereby reducing pressure on grasslands and playing a positive role in promoting grassland ecological protection and the development of herbivorous animal husbandry (3). Silage feed can preserve the nutritional characteristics of fresh forage for a long time, solve the seasonal imbalance of forage supply, and has advantages of good palatability, softness, and long storage time (3, 4). The nutritional components of silage corn are primarily composed of 25%−35% starch and 40%−50% neutral detergent fiber (NDF) (5). However, the harvested raw material has high moisture content and low crude protein (CP) content, making it prone to the proliferation of spoilage microorganisms, leading to a loss in dry matter (DM) content and making it difficult to meet the protein requirements of ruminant animals (6). The fermentation effectiveness and nutritional value of whole-plant corn silage are not always guaranteed. Hence, their enhancement has become a subject of extensive research. Numerous studies have established that incorporating microbial-enzyme additives specifically designed for silage effectively suppresses harmful bacteria while promoting beneficial populations, particularly lactic acid bacteria (LAB) (7, 8). In addition, the addition of unconventional feed resources rich in polyphenols can inhibit proteinase, reduce protein loss, and promote the ruminal digestion and utilization of silage forage (9, 10). Therefore, mixing whole-plant corn with other feed resources for silage or adding exogenous additives for silage is an effective scientific measure for preparing high-quality whole-plant corn silage feed.

Walnut (*Juglans regia L*.) is a plant in the Juglandaceae family, widely cultivated around the world (11). China has a long history of walnut cultivation, abundant resources, and ranks among the top in terms of planting area and production. Walnut green husk (WGH) also known as green dragon skin, refers to the thick green pericarp layer on the outside of the walnut, accounting for about 5/7 of the total weight of the walnut and is a byproduct of walnut processing (12). WGH is not only rich in nutrients such as amino acids, minerals, and dietary fiber but also contains bioactive components like flavonoids, polyphenols, and polysaccharides, which have functions such as antioxidant, antibacterial, and anti-inflammatory effects (11, 13).

Research shows that tannins, a type of plant polyphenol, can reduce the production of undesirable bacteria in silage and decrease dry matter loss, helping to improve the digestion and absorption of ruminants, enhance their production performance, and is one of the good material choices to improve silage quality (14). Tannin extracts have gained attention as functional silage additives for their ability to enhance the bacterial community and reduce nutrient loss. By also boosting animals' antioxidant and anti-inflammatory capacities, they represent a promising innovative feed material (15, 16).

Therefore, WGH can be used as an unconventional feed to mix with local forage for silage treatment. However, the effect of WGH supplementation on the fermentation quality and nutritional value of whole-plant corn silage remains to be investigated.

It was hypothesized that WGH would serve as a functional additive to enhance the fermentation quality and *in vitro* digestibility of whole-plant corn silage by favorably modulating its bacterial community. The present study was therefore conducted to evaluate the effects of different WGH inclusion rates on fermentation parameters, microbial population dynamics, and *in vitro* rumen fermentation characteristics. This research seeks to provide a scientific basis for improving silage quality and advancing the valorization of WGH.

## 2 Materials and methods

### 2.1 Raw materials and additives

WGH from the “Xinwen 185” variety was obtained in an air-dried form from Fengda Forestry and Agricultural Science and Technology Development Co., Ltd. (Aksu, Xinjiang). Whole-plant corn (“Qidan 828”) was harvested at the milk-ripening stage in September 2024 from Wushi County, Aksu Region, and was chopped into 1–2 cm segments. A commercial silage starter containing *Lactobacillus plantarum* (1.4 × 10^9^ CFU·g^−1^), *Lactobacillus buchneri* (6 × 10^8^ CFU·g^−1^), *xylanase* (2080 U·g^−1^), *cellulase* (336 U·g^−1^), and β*-glucanase* (1,920 U·g^−1^) was procured from the Beijing Precision Animal Nutrition Research Center.

### 2.2 Silage making

Four experimental treatments were established: a control (CK) consisting solely of whole-plant corn (Qidan 828). (1) Control group (CK); (2) A1 treatment + 15 g/kg of WGH; (3) A2 treatment + 30 g/kg of WGH; (4) A3 + 45 g/kg of WGH; the concentration of the additive was calculated according to the percentage of fresh matter (FM) of silage raw materials. These inclusion rates correspond to 44.4, 88.8, and 133.2 g·kg^−1^ on a dry matter basis, respectively, reflecting the significant difference in initial dry matter content between WGH (92.4%) and whole-plant corn (31.2%). A silage starter was dissolved in clean water (0.3 g per 150 ml) and uniformly applied to 10 kg of chopped forage. The treated materials were then vacuum-sealed in polyethylene bags (approximately 300 g per bag), with three replicates per treatment. All bags were stored in darkness at 25 °C for 60 days before being opened for analysis of fermentation quality, bacterial community structure, and *in vitro* rumen fermentation characteristics. The nutritional composition of raw materials in silage is indicated in Table 1.

**Table 1:** Nutritional composition of raw materials in silage (on a DM basis, %).

**Item**	**Whole-plant corn**	**WGH**
Dry matter (DM)	31.2	92.4
Crude ash (ash)	7.35	17.3
Crude protein (CP)	6.35	13.2
Water soluble carbohydrate (WSC)	10.2	7.54
Neutral detergent fiber (NDF)	51.7	50.3
Acid detergent fiber (ADF)	27.2	47.8

### 2.3 Silage quality analysis

Dry matter (DM) content was determined by weighing 300 g of silage samples were weighed and put in an air-forced oven at 105 to terminate enzymatic activity, followed by further drying at 65 °C for 48 hours until constant weight. The dried samples were ground through a 40-mesh sieve and analyzed for crude protein (CP) using the Kjeldahl method (17).

For fermentation analysis, 20 g of homogenized silage was blended with 180 ml of deionized water for 1 min. The filtrate was used to measure pH and ammonia nitrogen (NH_3_-N) content, while organic acids were quantified by high-performance liquid chromatography under the following conditions: C18 column (4.6 × 250 mm), 3 mmol·L^−1^ perchloric acid mobile phase at 0.8 ml·min^−1^, column temperature 30 °C, and detection wavelength 210 nm.

Microbial populations were assessed by homogenizing 10 g of silage with 90 ml of sterile physiological saline. Serial dilutions were plated on selective media for lactic acid bacteria, yeasts and molds, and coliforms, with colonies enumerated using the standard plate count method.

Aerobic stability was evaluated by monitoring temperature changes in silage samples exposed to air. The stability endpoint was defined as the time when silage temperature exceeded ambient temperature by 2 °C, recorded using multiple temperature probes per treatment.

### 2.4 Bacterial community analysis

Four groups of silage were sent to Personal Biotechnology Company (Nanjing, China) for bacterial diversity sequencing analysis. The total DNA of the samples was extracted using the PowerSoil^®^ DNA Isolation Kit (Thermo Fisher Scientific, Waltham, MA, USA). The V3–V4 hypervariable region of the bacterial 16S rRNA gene was amplified using fusion primers 338F (5′-ACTCCTACGGGAGGCAGCA-3′) and 806R (5′-GGACTACHVGGGTWTCTAAT-3′), which contained Illumina adapter sequences and sample-specific barcodes. Each sample was amplified in triplicate to ensure technical reliability during the PCR process. Illumina Tru Seq DNA was used to construct a sequencing library. Finally, Single Molecule Real-time (SMRT) sequencing of community DNA fragments was performed using the Pacbio Sequel third-generation sequencing platform.

### 2.5 *In vitro* fermentation test method

On the day of the experiment, rumen fluid was obtained from three donor sheep (12 months of age and BW of 45 ± 1.52 kg) at a slaughterhouse in Midong District, Urumqi. The donors' diet was designed according to NRC (2007) for finishing sheep (Table 2). Approximately 300–400 ml of ruminal fluid was collected from each animal, mixed to form a composite inoculum, and filtered through four layers of sterile cheesecloth. The filtrate was immediately transferred to a pre-sterilized thermos, which had been pre-warmed to 39 °C and filled with CO_2_, and was returned to the laboratory within 1 h. The artificial buffering solution was prepared following Menke et al. (18).

**Table 2:** Composition and nutrient levels of experimental diets (on a DM basis).

**Formula composition**	**Proportion %**	**Nutritional level**	**Content %**
Corn	26.0	Crude protein	11.5
Wheat bran	5.00	Ether extract	4.12
Cottonseed meal	12.0	Neutral detergent fiber	39.8
Rapeseed meal	2.50	Acid detergent fiber	24.0
Soybean meal	2.50	Ca	0.31
Premix	1.00	P	0.24
NaCl	0.50		
NaHCO_3_	0.50		
Wheat straw	50.0		
Total	100.0		

On day 60 of fermentation, silage samples were taken by opening the bag, and 0.5 g of dried and ground silage feed samples were weighed into fiber bags (triplicate), placed into the digestion tank of the DAISY II *in vitro* simulation incubator, and 1330 ml of A buffer (10 g/L potassium dihydrogen phosphate, 0.5 g/L magnesium sulfate, 0.5 g/L sodium chloride, 0.5 g/L calcium chloride, 0.5 g/L urea), 266 ml of B buffer (15 g/L sodium carbonate, 1 g/L sodium sulfide), and 400 ml of sheep rumen fluid collected before morning feeding were added. Carbon dioxide (CO_2_) was continuously injected into the digestion tank to maintain anaerobic conditions, and after the lid was sealed, the tank was placed into the *in vitro* simulation incubator. The samples were incubated at 39 °C for 48 h, then removed, and the fiber bags were rinsed with distilled water and dried. The *in vitro* dry matter digestibility (IVDMD) was calculated, and the remaining residue was used for nutrient digestibility determination. The fermentation fluid of the digestion tank was poured into a microcentrifuge tube (50 ml) and centrifuged (4 °C, 4500 rpm, 15 min) to collect the supernatant, used for measuring enzyme activity in the rumen.

#### 2.5.1 Analysis of nutritional digestibility

The post-fermentation residue from the *in vitro* rumen incubation was analyzed for dry matter (DM), crude protein (CP), neutral detergent fiber (NDF), and acid detergent fiber (ADF) contents according to the standard procedures of AOAC (19). Nutrient digestibility was then calculated using the following formula:

Digestibility of a nutrient in silages = [(nutrient content before fermentation – nutrient content after fermentation)/nutrient content before fermentation] × 100%

#### 2.5.2 Analysis of rumen enzyme activity

Enzyme activities in the rumen fluid, including carboxymethyl cellulase, cellobiase, amylase, pectinase, protease, and xylanase, were determined using commercial assay kits (Shanghai Personal Biotechnology Co., Ltd., China). The measurements were conducted following the manufacturer's protocols, and the absorbance was read with a ChroMate^®^ 4300 microplate reader (Awareness Technology, Inc., USA).

### 2.6 Statistical analysis

All experimental data, except for microbial sequencing data, were expressed as means and analyzed using one-way analysis of variance (ANOVA) in SPSS software (version 26.0). Significant differences among treatment means were determined by Duncan's multiple range test at a significance level of *P* < 0.05. Orthogonal polynomial contrasts were applied to evaluate the linear and quadratic effects of the WGH inclusion level. The high-throughput sequencing data of the bacterial community were processed and analyzed on the Genes Cloud online platform (https://www.genescloud.cn).

## 3 Results

### 3.1 Silage quality

As shown in Table 3, WGH inclusion significantly improved silage quality. DM and CP content increased linearly (*P* < 0.05), while LA, AA, PA, NH_3_-N, and yeast counts decreased linearly (*P* < 0.05). The CK group had lower CP, pH, and LAB counts than other treatments (*P* < 0.05). The A3 group showed higher DM but lower LA, AA, and PA than other groups (*P* < 0.05). Butyric acid was undetected. NH_3_-N and yeast count in WGH-treated groups were lower than CK (*P* < 0.05). Additionally, DM, CP, pH, and LAB counts increased linearly with WGH level (*P* < 0.01), while LA, AA, PA, NH_3_-N, and yeast counts decreased linearly (*P* < 0.01). CP, LA, AA, PA, NH_3_-N, and aerobic stability showed quadratic effects (*P* < 0.01).

**Table 3:** Effect of walnut green husk on the fermentation quality of whole-plant corn silage.

**Item**	**Treatment**				**P**		
	**CK**	**A1**	**A2**	**A3**	**Group**	**Linear**	**Quadratic**
DM/%FM	37.8^b^	38.4^b^	39.1^b^	40.3^a^	<0.01	<0.01	0.15
CP/%DM	7.15^b^	7.82^a^	8.03^a^	8.19^a^	<0.01	<0.01	<0.01
pH	3.81^c^	3.87^b^	3.90^b^	4.00^a^	<0.01	<0.01	0.21
LA/%DM	10.1^a^	9.56^b^	9.01^c^	8.16^d^	<0.01	<0.01	<0.01
AA/%DM	2.01^a^	1.92^b^	1.67^c^	1.03^d^	<0.01	<0.01	<0.01
PA/%DM	0.71^a^	0.66^b^	0.65^b^	0.46^c^	<0.01	<0.01	<0.01
BA/%DM	ND	ND	ND	ND	ND	ND	ND
NH_3_-N/%TN DM	3.26^a^	3.13^b^	2.23^c^	2.03^d^	<0.01	<0.01	<0.01
Lactic acid bacteria (Ig CFU/g)	6.78^c^	6.85^b^	6.98^a^	7.03^a^	<0.01	<0.01	0.71
Yeast (Ig CFU/g)	3.53^a^	3.45^b^	3.39^c^	3.36^c^	<0.01	<0.01	0.17
Mold (Ig CFU/g)	ND	ND	ND	ND	ND	ND	ND
Aerobic stabilization time/h	117.3^c^	122.7^a^	125.7^ab^	121.3^bc^	<0.01	0.13	<0.01

Data in the same column with different small letters indicate a significant difference (P < 0.05); CK: control group; A1: addition of 15 g·kg^−1^ walnut green husk, A2: addition of 30 g·kg^−1^ walnut green husk, A3: addition of 45 g·kg^−1^walnut green husk. The contents of dry matter (DM), crude protein (CP), lactic acid (LA), Acetic acid (AA), propionic acid (PA), and butyric acid (BA) were determined.

### 3.2 Bacterial community

**Figure 1:**
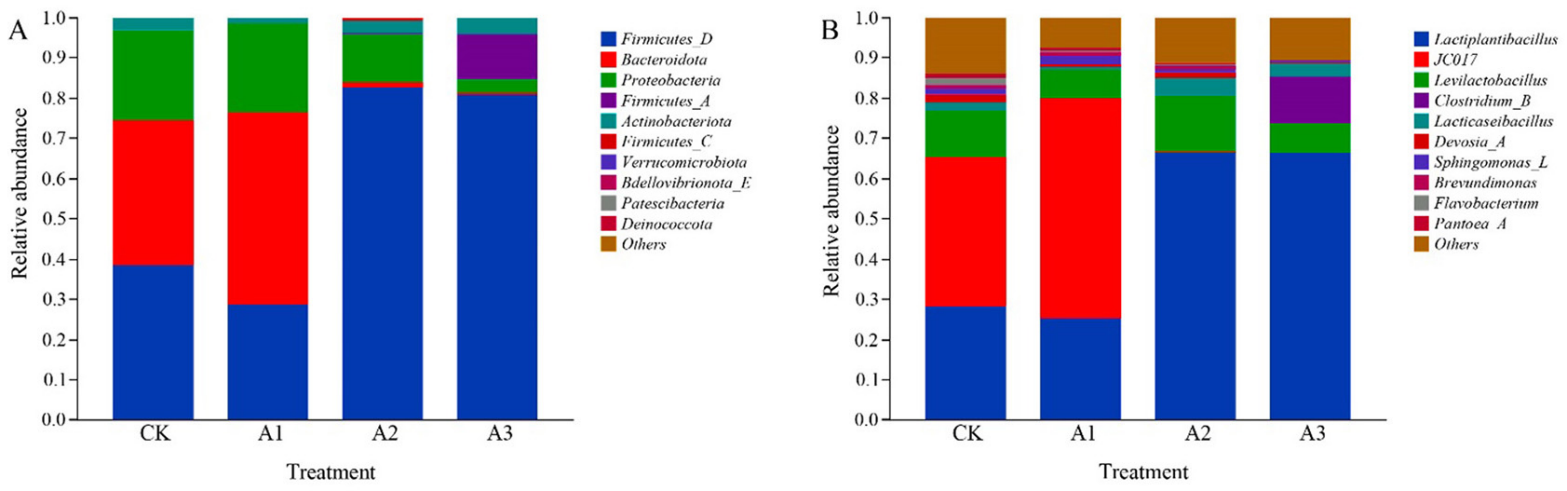
Relative abundance of species at phylum level **(A)** and genus level **(B)** of different forage silage treatment groups.

At the phylum level (Figure 1A), *Firmicutes_D, Bacteroidota*, and *Proteobacteria* were identified as the dominant phyla (relative abundance >1%) across all treatments. Their relative abundances in the CK, A1, A2, and A3 groups were as follows: *Firmicutes_D* (38.68%, 28.79%, 82.79%, and 80.88%), *Bacteroidota* (35.74%, 47.88%, 1.21%, not applicable), and *Proteobacteria* (22.62%, 21.85%, 12.10%, and 3.36%). Additionally, *Firmicutes_A* represented 11.41% in group A3.

At the genus level (Figure 1B), the dominant genera (relative abundance >1%) exhibited clear shifts with WGH treatment. In the CK and A1 groups, the dominant genera were *Lactiplantibacillus* (28.44%, 25.37%), *JC017* (37.04%, 54.61%), and *Levilactobacillus* (11.31%, 7.14%). In contrast, the A2 group was dominated by *Lactiplantibacillus* (66.48%), *Levilactobacillus* (14.03%), and *Lacticaseibacillus* (4.22%), while the A3 group was characterized by *Lactiplantibacillus* (66.33%), *Clostridium_B* (11.62%), and *Levilactobacillus* (7.30%).

### 3.3 Bacterial α diversity and β diversity

**Figure 2:**
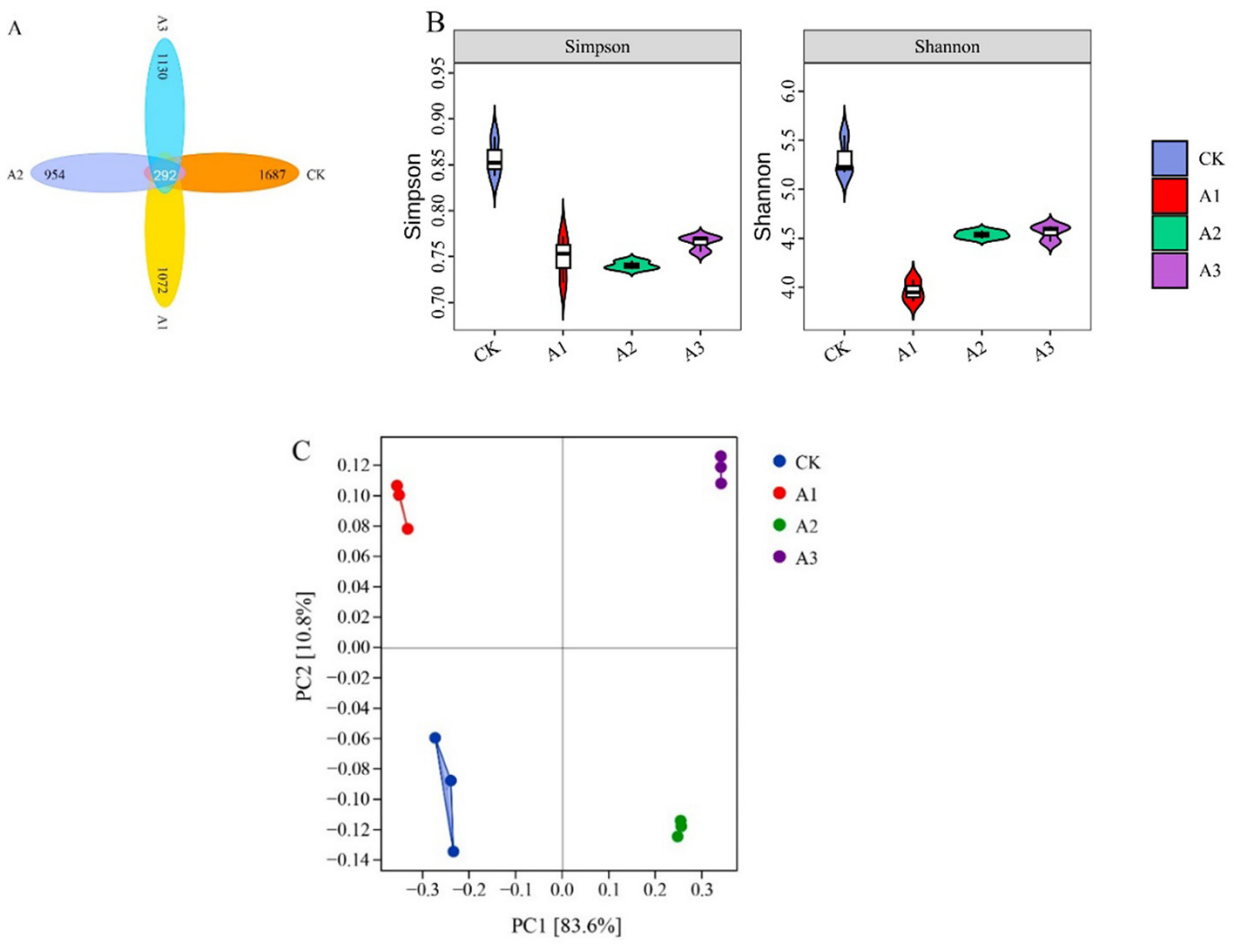
Alpha diversity and Bata diversity analysis of different forage silage treatment groups. Venn analysis of the operational taxonomic units (OTUs) in the silage samples from the different combinations **(A)**. The variations in community alpha-diversities (Simpson index and Shannon index) **(B)**. The community dissimilarities in Differences in the addition amount of different walnut green husk, calculated via bray curtis distances, with coordinates calculated using principal coordinates analysis (PCoA) **(C)**. CK, control group (Whole-plant corn); A1, addition of 15 g·kg^−1^ walnut green husk; A2, addition of 30 g·kg^−1^ walnut green husk; A3, addition of 45 ·kg^−1^ walnut green husk.

The total number of OTUs detected across the four groups of silage feed was 5135, with a total of 292 OTUs, accounting for 5.69% (Figure 2A). The number of OTUs in the CK, A1, A2, and A3 treatment groups was 1,687, 1,072, 954, and 1,130, respectively. The Simpson and Shannon indices for the four treatment groups are shown in Figure 2B, indicating that the Simpson and Shannon indices of the walnut pericarp treatment groups were both lower than those of the CK treatment group. Notably, the Simpson index significantly decreased under the A2 treatment, while the Shannon index significantly decreased under the A1 treatment. The PCoA analysis based on Bray-Curtis distance (Figure 2C) showed that samples with more similar composition clustered more closely in the PCA plot, with PC1 and PC2 accounting for 83.6 and 10.8%, respectively. The bacterial communities of the four treatment groups were distinctly separated, with each group's samples clustering well.

### 3.4 Bacterial LEfSe species differentiation

**Figure 3:**
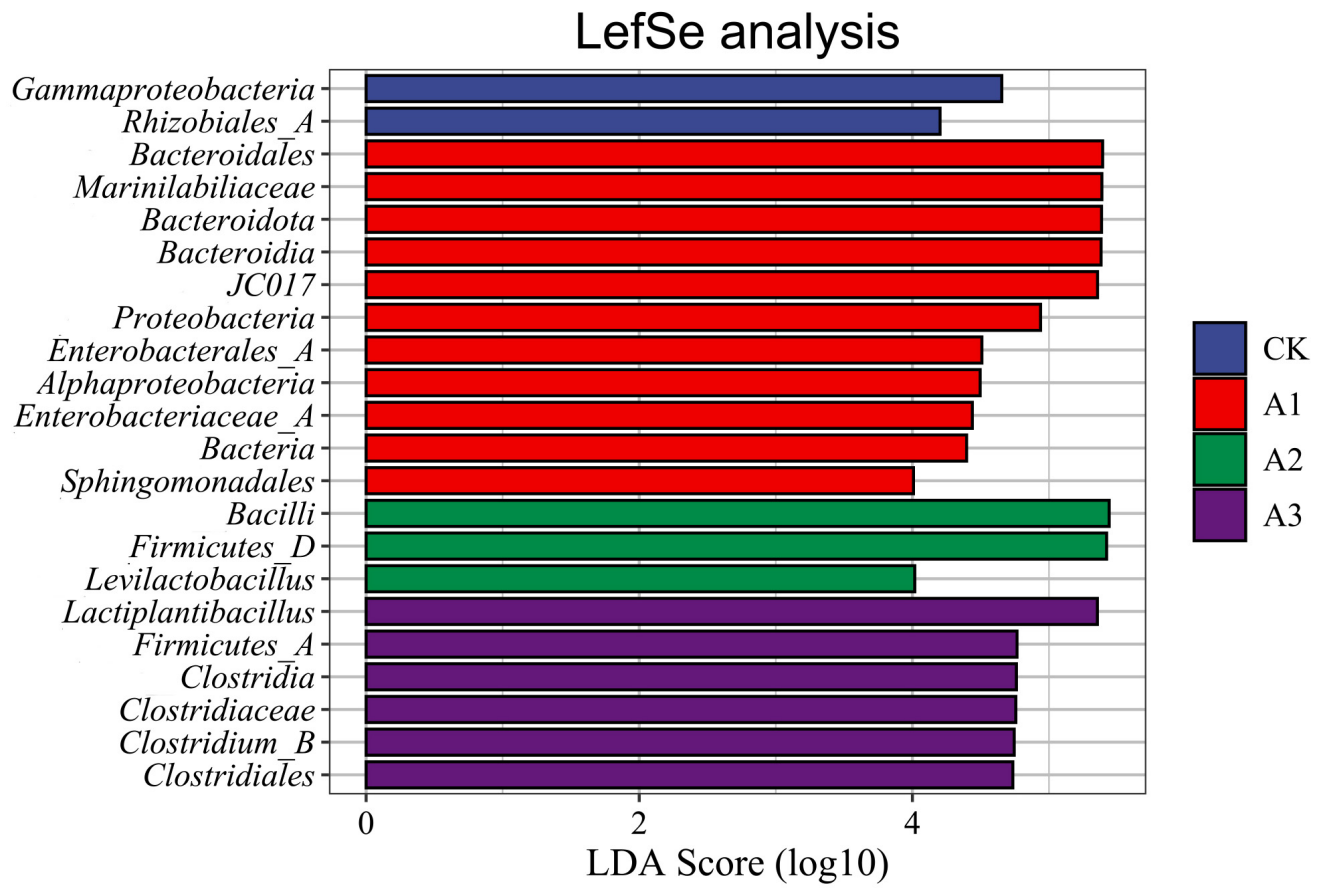
Linear discriminant analysis of different forage silage treatment groups.

Through LDA analysis of the bacterial community in silage (LDA > 2, *P* < 0.05), Figure 3 shows that there were significant differences in intergroup abundance for four phyla, five classes, five orders, three families, and four genera. The significantly enriched species at the phylum level in the CK, A1, A2, and A3 groups were *Bacteroidota, Proteobacteria, Firmicutes_D*, and *Firmicutes_A*, respectively; at the class level, they were *Gammaproteobacteria, Bacteroidia, Alphaproteobacteria, Bacilli*, and *Clostridia*; at the order level, they were *Rhizobiales_A, Bacteroidales, Enterobacterales_A, Sphingomonadales*, and *Clostridiales*; at the family level, they were *Marinilabiliaceae, Enterobacteriaceae_A*, and *Clostridiaceae*; and at the genus level, they were *JC017, Levilactobacillus, Lactiplantibacillus*, and *Clostridium_B*.

### 3.5 *In vitro* nutrient digestibility

**Figure 4:**
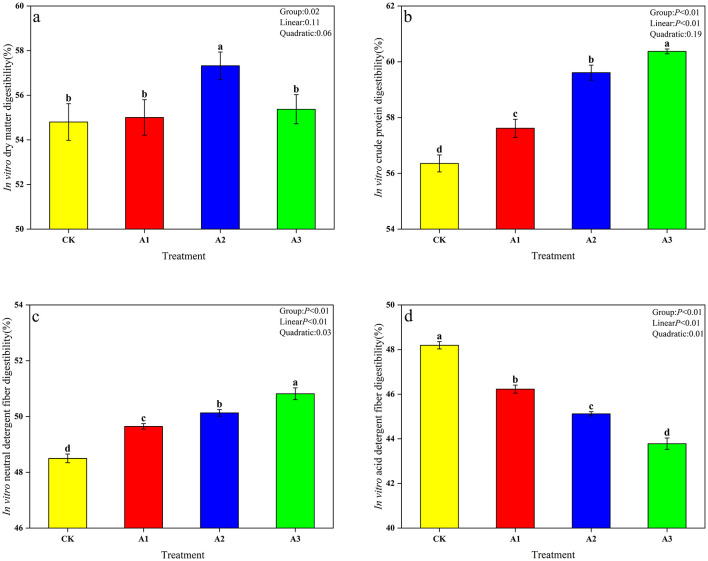
Effect of walnut green husk on in vitro nutrient digestibility of whole-plant corn silage. Representative photograph of *In vitro* dry matter digestibility **(a)**. *In vitro* crude protein digestibility **(b)**. *In vitro* neutral detergent fiber digestibility **(c)**. *In vitro* acid detergent fiber digestibility **(d)**. Different lowercase letters marked on the data column indicate significant difference (*P* < 0.05). CK, control group (Whole-plant corn); A1, addition of 15 g·kg^−1^ walnut green husk; A2, addition of 30 g·kg^−1^ walnut green husk; A3, addition of 45 g·kg^−1^ walnut green husk.

The IVDMD content first increased and then decreased with the increase in the addition ratio of WGH, while the IVCPD and IVNDFD content gradually increased and the IVADFD content gradually decreased (Figure 4). Among them, the IVDMD content of group A2 was significantly higher than that of other treatment groups (*P* < 0.05); the IVCPD and IVNDFD content of group A3 were significantly higher than those of other treatment groups (*P* < 0.05), while the IVADFD content was significantly lower than that of other treatment groups (*P* < 0.05). Additionally, increasing the WGH inclusion linearly increased the IVCPD and IVNDFD (*P* < 0.05) but decreased the IVADFD (*P* < 0.05), with all three parameters showing significant quadratic effects (*P* < 0.05).

### 3.6 Rumen digestive enzyme

**Figure 5:**
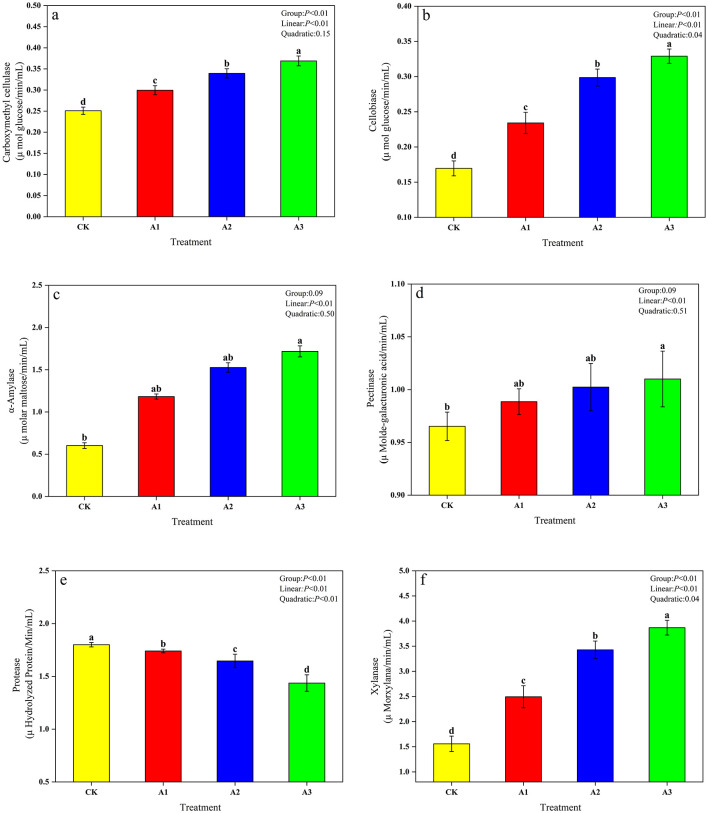
Effect of walnut green husk on rumen digestive enzymes of whole-plant corn silage. Representative photograph of Carboxymethyl cellulase **(a)**. Cellobiase **(b)**. α-Amylase **(c)**. Pectinase **(d)**. Protease **(e)**. Xylanase **(f)**. Different lowercase letters marked on the data column indicate significant difference (*P* < 0.05). CK, control group (Whole-plant corn); A1, addition of 15 g·kg^−1^ walnut green husk; A2, addition of 30 g·kg^−1^ walnut green husk; A3, addition of 45 g·kg^−1^ walnut green husk.

Rumen digestive enzyme activities responded differentially to WGH supplementation. The activities of carboxymethyl cellulase, cellobiase, amylase, pectinase, and xylanase were enhanced, whereas protease activity was suppressed with increasing WGH levels (Figure 5). The protease activity in the CK group was significantly higher than that in other treatment groups (*P* < 0.05), while the activities of carboxymethyl cellulase, Cellobiase, and xylanase were significantly lower than those in other treatment groups (*P* < 0.05); the activities of α-amylase and pectinase in the A3 group were significantly higher than those in the CK group (*P* < 0.05). Additionally, with the increase in the addition ratio of WGH, the protease activity in whole corn silage in the rumen showed a linear decrease (*P* < 0.05), while the activities of other rumen digestive enzymes showed a linear increase (*P* < 0.05). Cellobiase, protease, and xylanase activity exhibited significant quadratic curve effects (*P* < 0.05).

## 4 Discussion

### 4.1 Silage quality

The suitable DM content for silage ranges from 28 to 40% (20). In this experiment, the DM content of whole-plant corn silage ranged from 37.75 to 40.32%, falling within the optimal recommended range. Furthermore, as the WGH addition rate increased, the DM content demonstrated an increasing trend, indicating that WGH plays a crucial role in reducing moisture content, effectively improving dry matter recovery (DMR) and minimizing nutrient loss. Dunière et al. (21) demonstrated that silage with higher DMR and lower moisture content exhibits a reduced maximum temperature increase during aerobic exposure, prolongs the time before exceeding ambient temperature by 2 °C, and thereby enhances aerobic stability. Compared to whole-plant corn silage alone, the aerobic stability time significantly improved following WGH addition, consistent with the conclusions drawn by Dunière et al. (21). Silage fermentation inevitably leads to CP loss, directly reducing the nutritional value of silage and adversely affecting ruminant nutrition (22). Pathogenic bacteria such as clostridia and enterobacteria degrade proteins in silage into NH_3_-N, which is further broken down by harmful microorganisms, leading to nitrogen loss through volatile compounds like ammonia. Therefore, NH_3_-N content reflects the extent of protein degradation in silage (23). In this study, as the WGH proportion increased, the CP content gradually improved while NH_3_-N content decreased, demonstrating effective inhibition of protein loss in whole-plant corn silage. This may be attributed to tannins in WGH forming complexes with proteins, reducing NH_3_-N generation and preventing CP degradation, aligning with the findings of Wang et al. (17).

pH serves as an intuitive indicator of silage quality, with high-quality silage typically exhibiting a pH below 4.2 (24). In this study, the pH of both whole-plant corn and WGH-mixed silage remained below 4.2, meeting the standards for high-quality silage. This indicates that WGH addition did not disrupt the normal fermentation process, allowing successful co-fermentation of the two materials. The content and composition of organic acids are critical metrics for evaluating silage quality (25). According to Yang et al. (26), high-quality silage should contain over 3% LA, 1%−4% AA, and nearly 0% BA on a dry matter basis. In this experiment, all treatments met these criteria for LA, AA, and BA content. The WGH treatment group exhibited lower organic acid levels than alfalfa silage, supporting the hypothesis that tannin-rich materials can inhibit the growth of organic acid producing bacteria (27). The abundance of lactic acid bacteria (LAB) is a key determinant of fermentation efficiency during silage processing. In this study, LAB counts increased linearly with WGH addition, all exceeding 5.0 lg CFU·g^−1^, meeting the standard for well-preserved silage. This suggests that WGH promotes LAB proliferation. During silage fermentation, yeasts metabolize carbohydrates, producing ethanol and carbon dioxide. Excessive ethanol production reduces silage palatability (28). The yeast count in whole-plant corn silage was higher than in WGH-treated groups, possibly due to the higher WSC content in whole-plant corn providing more substrates for yeast growth, consistent with the conclusion of Oliveira et al. (29). During aerobic exposure, prolonged air contact leads to substantial fungal growth, accompanied by a gradual temperature increase in silage. Yeast populations are considered the primary drivers of aerobic fungal proliferation (30). In this study, WGH addition resulted in a linear decrease in yeast counts in whole-plant corn silage, accompanied by a slower temperature rise during aerobic exposure and an extended aerobic stability period compared to whole-plant corn silage alone. These findings are consistent with the research results of Chen et al. (31).

### 4.2 Bacterial community

Bacteria play a crucial role in silage fermentation, and analyzing the bacterial community structure and relative abundance enhances our understanding of fermentation efficiency (32). At the phylum level, the dominant bacteria across all treatment groups were *Firmicutes_D, Bacteroidota*, and *Proteobacteria*, consistent with Yin et al.'s (33) findings on the predominant phyla in whole-plant corn silage. *Firmicutes* encompass beneficial groups such as LAB and spore-forming bacteria, which decompose organic matter into substrates more readily utilized by LAB, exhibiting capabilities in cellulose and protein degradation to improve feed nutritional value (34). In this study, the proportion of *Firmicutes_D* significantly increased in the A2 and A3 treatments, indicating its pivotal role in modulating the bacterial community structure and enhancing the fermentation quality of whole-plant corn silage. The increasing trend in *Firmicutes* relative abundance with higher WGH supplementation paralleled the linear increase in LAB counts, aligning with Xu et al.'s (35) report that elevated *Firmicutes* abundance corresponds to increased LAB populations. *Bacteroidota* primarily participate in hydrolyzing macromolecular organic matter in silage, decomposing carbohydrates into small molecules such as LA and AA (36). Correspondingly, this study observed a declining trend in *Bacteroidota* relative abundance with increasing WGH addition, accompanied by gradual reductions in LA and AA content. Furthermore, the relative abundance of *Proteobacteria* decreased progressively with higher WGH proportions, while CP content significantly increased. This supports Silva et al.'s (37) conclusion that *Proteobacteria* in silage can generate ammonia via deamination, leading to reduced CP content. Thus, an increase in *Proteobacteria* relative abundance is detrimental to efficient protein utilization in silage. At the genus level, the predominant bacteria across the four treatment groups were *Lactiplantibacillus, Levilactobacillus*, and *Lacticaseibacillus*. These dominant genera may represent key microorganisms regulating quality formation during co-fermentation of WGH and whole-plant corn silage, corroborating Yang et al.'s findings (38). The increased relative abundance of *Lactiplantibacillus* following WGH supplementation suggests its beneficial role in modulating LAB production and improving fermentation efficacy, consistent with Peng et al.'s (39) study on LAB and sea buckthorn residue in Paulownia tomentosa silage. *Levilactobacillus*, a Gram-positive bacterium with a pH tolerance range of 4.0–8.0, has been confirmed to decompose monosaccharides into organic acids, effectively promoting pH reduction (40). In this experiment, the degradation and consumption of high WSC content in whole-plant corn silage resulted in the lowest pH, supporting this conclusion. The highest relative abundance of *Levilactobacillus* under the A2 treatment indicates that 30 g·kg^−1^ WGH effectively enhances the nutritional value of whole-plant corn silage and inhibits spoilage bacteria proliferation (41). Similarly, the increased relative abundance of *Lacticaseibacillus* and significantly improved aerobic stability following WGH addition suggest its role as a beneficial genus that suppresses spoilage microorganisms and enhances silage stability.

Numerous studies have demonstrated that microbial community composition and structure are largely regulated by the ensiling process, with successful fermentation often associated with reduced microbial diversity (42, 43). Operational taxonomic units (OTUs), Shannon index, and Simpson index are commonly used to evaluate microbial α-diversity, reflecting community diversity and richness (44). In this study, WGH supplementation significantly reduced the OTUs, Shannon index, and Simpson index of whole-plant corn silage, indicating decreased microbial diversity and richness. This may be attributed to tannins in WGH promoting LAB dominance during fermentation, increasing the relative abundance of specific beneficial bacteria while inhibiting other bacterial communities. β-diversity analysis revealed structural changes in bacterial communities of WGH-mixed silage, with principal coordinates analysis (PCoA) highlighting overall inter-sample distances, consistent with ecological data characteristics (45). Significant separation of bacterial communities among the four treatment groups confirms that varying WGH levels substantially influence microbial composition, suggesting that WGH shapes the bacterial community structure in whole-plant corn silage, leading to divergent fermentation outcomes and quality characteristics.

It should be acknowledged that the sample size (*N* = 3) for high-throughput sequencing and *in vitro* analysis, while adequate for detecting major treatment effects, represents a limitation in capturing the full complexity of microbial community dynamics and digestion parameters.

### 4.3 *In vitro* rumen digestion characteristics

Nutrient digestibility serves as a key indicator of silage utilization in the ruminant digestive system. An increase in IVDMD enhances silage digestibility and is positively correlated with feed intake in ruminants (46). In this experiment, WGH supplementation significantly improved IVDMD, likely attributable to alterations in the fermentation substrate for rumen microorganisms. The concurrent increase in carboxymethyl cellulase activity promoted the degradation of cellulose and hemicellulose components, thereby enhancing enzyme-substrate interactions and improving silage digestibility. This process reduces the loss of digestible nutrients and contributes to the overall nutritional value of silage (47). The IVCPD of silage is influenced by multiple factors including CP content, protease activity, and rumen retention time. The gradual increase in IVCPD with higher WGH proportions observed in this study can be attributed to the high CP content of WGH and, importantly, to the beneficial reduction in protease activity. This decrease in protease activity represents a key protective mechanism rather than an undesirable effect, as it minimizes protein degradation in the rumen, reduces ammonia nitrogen production, and enhances the efficiency of dietary protein utilization. This suggests that tannins in WGH selectively inhibit proteolytic bacteria in the rumen, reduce protease secretion, and exert a protective effect on proteins, thereby effectively enhancing IVCPD. IVNDFD and IVADFD reflect the ruminal degradability of fibrous components in silage (48). NDF comprising cellulose, hemicellulose, and lignin, represents the most abundant yet recalcitrant carbohydrate polymers in plant cell walls (49). The degradation of these components depends on specific enzymatic activities: carboxymethyl cellulase targets cellulose chains, xylanase hydrolyzes hemicellulose backbones, and pectinase facilitates cell wall disintegration. Consequently, digestive enzyme activity directly determines IVNDFD levels. In this study, increasing WGH proportions enhanced the activities of carboxymethyl cellulase, pectinase, and xylanase, thereby promoting IVNDFD in a proportional relationship. Conversely, IVADFD exhibited a gradual decline with increasing WGH addition, potentially due to the higher acid detergent fiber (ADF) content in WGH compared to whole-plant corn, combined with the limited lignin degradation capacity of rumen microorganisms and extended lignin retention in the rumen. These findings highlight the importance of disrupting plant cell wall structures to reduce their resistance to microbial attack, increase the degradable fraction in mixed silage, and ultimately improve fiber digestibility.

Furthermore, WGH supplementation enhanced the activities of most digestive enzymes except protease. This differential effect may be explained by the selective inhibition of proteolytic rumen microorganisms by polyphenolic tannins, reducing protease secretion. This selective reduction in protease activity, while maintaining or enhancing other enzymatic functions, demonstrates the targeted mechanism through which WGH tannins modulate rumen fermentation. The consistent suppression of protease across all WGH treatment levels provides compelling evidence for tannin-mediated protein protection in the rumen environment. Conversely, these tannins might stimulate the proliferation of fiber-degrading bacteria, increasing the production of cellulases (carboxymethyl cellulase, cellobiase), hemicellulases (xylanase), and pectinase.

## 5 Conclusion

This study demonstrates that WGH significantly improves whole-plant corn silage quality by increasing DM and CP content, enhancing lactic acid bacteria populations, while reducing yeast counts, NH_3_-N and PA content. *In vitro* rumen fermentation analysis showed that with increasing WGH levels, IVDMD, IVCPD, and IVNDFD significantly increased while IVADFD decreased. The activity of all digestive enzymes except protease was enhanced, improving overall nutrient digestibility. WGH supplementation reduced bacterial community diversity but optimized its structure. The A2 treatment (30 g·kg^−1^ WGH) resulted in the highest relative abundance of *Lactiplantibacillus, Lacticaseibacillus*, and *Peptidiphaga*, along with significantly improved aerobic stability. Based on these comprehensive results, the addition of 30 g·kg^−1^ WGH (A2 group) is recommended as the optimal level for enhancing whole-plant corn silage quality, ruminal digestibility, and bacterial community structure.

## Data Availability

The data presented in the study are deposited in the NCBI Sequence Read Archive (SRA) repository, accession number PRJNA1397887 (https://www.ncbi.nlm.nih.gov/bioproject/PRJNA1397887).
